# Effect of Ebenatide on glycemic metabolism and body fat in patients with type 2 diabetes mellitus

**DOI:** 10.3389/fendo.2025.1622526

**Published:** 2025-06-18

**Authors:** Cheng-lan Xu, Xiao-cen Kong, Xiao-mei Liu, Xiao-hua Xu, Bing-li Liu, Jian-hua Ma

**Affiliations:** Department of Endocrinology, Nanjing First Hospital, Nanjing Medical University, Nanjing, China

**Keywords:** Ebenatide, type 2 diabetes mellitus, GLP-1, continuous glucose monitoring, body fat

## Abstract

**Aim:**

Investigate effects of Ebenatide, a novel glucagon-like peptide-1 analogue, on glycemic control and body composition in type 2 diabetes mellitus (T2DM).

**Methods:**

This randomized, prospective, interventional study enrolled 78 subjects (76 finished). Subjects were randomized to either the Ebenatide group (52 subjects, Ebenatide for 52 weeks) or the placebo group (24 subjects, placebo for 24 weeks followed by Ebenatide for 28 weeks) according to a 2:1 allocation ratio. Assessments included continuous glucose monitoring and body composition analysis.

**Results:**

The Ebenatide group showed significantly lower in hemoglobin A1c (HbA1c), mean blood glucose (MBG), time above range (TAR) and standard deviation (SD), along with improvement of time in range (TIR) at Week 24 and Week 52 compared to baseline (*P*<0.05). The triglyceride-glucose index (TyG) decreased at Week 52 compared with baseline (*P*<0.01). Compared with the placebo group, the Ebenatide group demonstrated greater reductions in HbA1c and TAR and improved TIR at Week 24 (*P*<0.05), but no difference at Week 52. Body composition analysis showed that the Ebenatide group had significant declines in weight, BMI, body fat and waist-to-height ratio (WHtR) (*P*<0.05). Compared with baseline, the Ebenatide group exhibited decreased blood pressure and hemoglobin levels and elevated serum amylase and lipase levels at Week 24 and Week 52 (*P*<0.05). Adverse events were limited to gastrointestinal reactions.

**Conclusion:**

Ebenatide treatment for 24 weeks significantly improved HbA1c, TIR, TyG, weight and WHtR in T2DM subjects, with these benefits sustained for at least 52 weeks.

**Clinical trial registration:**

https://clinicaltrials.gov/, identifier NCT05990374.

## 1 Introduction

With the gradual aging of the population structure, the prevalence of type 2 diabetes mellitus (T2DM) has nearly doubled in the past decade, reaching 12.8% in China (1) while mortality rates show an upward trend (2). In clinical treatment, in addition to strict glycemic control, the protective effects of medications on the cardiovascular system, kidneys, and other organs have gained increasing attention. Glucagon-like peptide-1 receptor agonists (GLP-1RAs), which can simultaneously regulate blood glucose and body weight (3) while offering cardiovascular and cerebrovascular protection (4), are now widely recommended in T2DM treatment (5).

Ebenatide, a novel drug, is derived from lizard-origin Exendin-4. As a once-weekly long-acting GLP-1RA, Ebenatide potentially prolongs its half-life beyond some current analogs. Ebenatide is modified through a proprietary drug affinity complex technology, enabling it forms a stable Exendin-4-albumin conjugate. This structure resists quickly degradation by dipeptidyl peptidase-4 (DPP-4) and avoids rapid renal clearance. Its albumin-binding moiety may enhance tissue distribution while reducing renal clearance, a pharmacokinetic profile distinct from either short- or long-acting GLP-1RAs.

Continuous Glucose Monitoring (CGM) comprehensively evaluates glycemic control through multidimensional parameters. In 2017, an international consensus statement on CGM core metrics was released, recognized CGM-related indices as important tools for assessing glycemic levels in patients with T2DM (6). Among these, Time in Range (TIR) has emerged as a new glycemic control metric alongside hemoglobin A1c (HbA1c) and is associated with diabetes-related complications (7).

Body composition assessment through bioelectrical impedance analysis offers a convenient, noninvasive approach that yields additional physiological parameters compared to conventional anthropometric measurements (8). Adiposity and muscle-related indices not only assess obesity and metabolic risk but also reflect muscle functional status, providing critical evidence for nutritional interventions, exercise rehabilitation, and chronic disease management.

In this study on Ebenatide, in addition to conventional efficacy assessments such as fasting blood glucose (FBG), HbA1c, body mass index (BMI), and waist circumference (WC), more precise and clinically significant indicators—including CGM, body composition analysis, triglyceride-glucose index (TyG), and waist-to-height ratio (WHtR)—were employed to further evaluate the drug’s therapeutic effects.

Studies have demonstrated that WHtR exhibits stronger correlation with T2DM than conventional anthropometric indices including waist-to-hip ratio and BMI (9). Emerging evidence suggests WHtR may serve as a superior predictor for cardiovascular risk assessment (10). The TyG, derived from FBG and triglyceride (TG), has gained increasing recognition in recent years for its utility in evaluating insulin resistance of T2DM (11). This index has also been proposed as a potential predictor for diabetic kidney disease (12). Notably, elevated TyG levels have been associated with higher risks of coronary artery disease, progression of heart failure, and both incidence and recurrence of ischemic stroke (13, 14). In the present study, we employed this index to further evaluate the potential effects of Ebenatide on insulin resistance improvement.

This study fills the gap in evaluating the therapeutic efficacy of Ebenatide. These innovative assessment strategies may provide a more comprehensive understanding of the metabolic health impacts of GLP-1RAs and may offer new evidences for the standardized clinical use of GLP-1RAs.

## 2 Methods

### 2.1 Participants

We designed the trial with primary endpoint defined as glycated hemoglobin (HbA1c) at 24 weeks. Based on an assumed population standard deviation of 1.2% and an expected between‐group difference of 1% in HbA1c, the smallest difference that we considered to be clinically meaningful, we set a two‐sided significance level (α) of 0.05 and statistical power of 80%. Under a 2:1 allocation ratio, the initial calculations indicated a requirement of 36 participants in the treatment arm and 18 in the placebo arm. To allow for anticipated dropout rates of 30% in the treatment arm and 20% in the placebo arm, the final target sample sizes were increased to 52 and 24, respectively. All sample‐size calculations were performed using the R package pwr.

Between August 2023 and September 2024, a total of 78 patients with T2DM were recruited as inpatients at the Department of Endocrinology, Nanjing First Hospital. The inclusion criteria included patients aged 18–70 years with 20 kg/m²≤ BMI ≤40 kg/m², and 7.0%≤ HbA1c ≤11.0%. The study population included both treatment-naïve patients with newly diagnosed T2DM and patients who had received stable maximal tolerated doses of metformin for ≥3 months prior to enrollment. The exclusion criteria were (1): hyperglycemia: from randomization to Week 6: FBG >15 mmol/L, from the Week 7 to Week 12: FBG >13.3 mmol/L, from Week 13 to the Week 24: FBG >11.1 mmol/L (200 mg/dL), from Week 25 to Week 52: FBG >11.1 mmol/L (2). history of long-term use of medications affecting gastrointestinal motility, and use of weight-loss drugs or hormonal therapy within the past 3 months (3). impaired liver/kidney function: alanine aminotransferase and/or aspartate aminotransferase >2.5×ULN or total bilirubin >1.5×ULN; estimated glomerular filtration rate <60 mL/min; systolic blood pressure (SBP) >160 mmHg and/or diastolic blood pressure (DBP) >100 mmHg; fasting triglycerides >5.64 mmol/L; serum amylase or lipase >3×ULN (4). history of gastric-related surgeries (5). history of pancreatitis, medullary thyroid carcinoma, or multiple endocrine neoplasia type 2 (6). recent weight-loss attempts or pregnancy plans.

All participants on metformin pre-enrollment continued their stable doses throughout the study, with no dosage adjustments permitted unless medically necessary.

### 2.2 Experimental procedure

This was a randomized, prospective, interventional study (Clinical Trial Register identifier NCT05990374). The study protocol was approved by the institutional ethical committee of Nanjing First Hospital, Nanjing Medical University. Informed consent was provided by all recruited subjects. All procedures followed were in accordance with the Helsinki Declaration of 1964, as revised in 2013.

**Figure 1:**
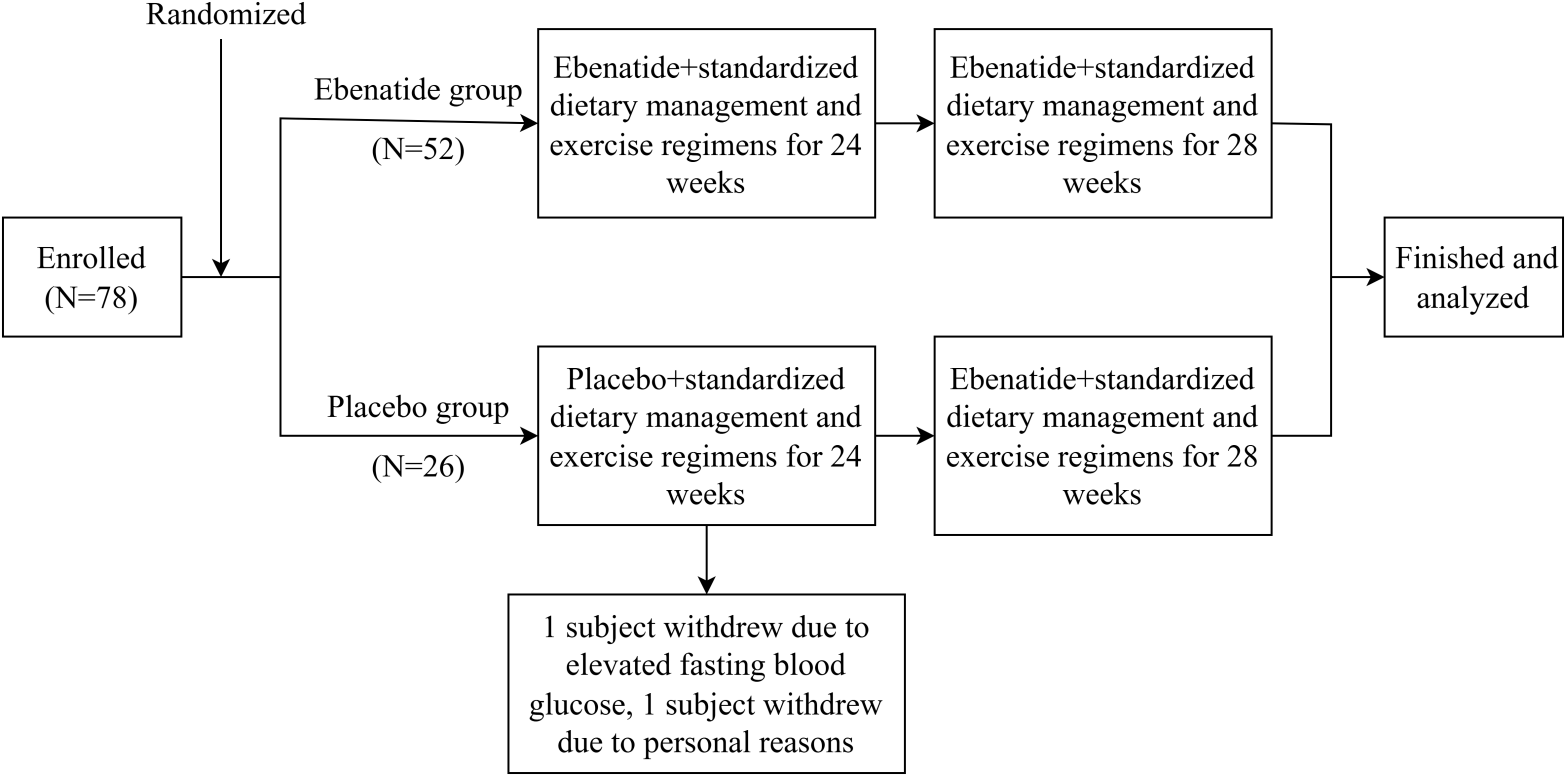
Study flow chart.

A stratified block randomization was employed, with metformin usage (yes/no) as the stratification factor. The allocation sequence was generated and concealed through a centralized interactive web-response system (IWRS). Participants were randomized at a 2:1 ratio to either Ebenatide (n=52) or placebo (n=26) group, with a fixed block size of 6 to ensure inter-group balance. This study utilized a double-blind design, where the investigational drug and placebo were identical in appearance, packaging, and odor. Both participants and investigators remained blinded to group assignments until database lock. The study flowchart is shown in **Figure 1**.

All subjects continued structured medical nutrition therapy (MNT) and moderate-intensity physical activity throughout the study, as per ADA guidelines (15).

Both Ebenatide and placebo were provided by Changshan Kaijiejian Biopharmaceutical R&D Co., Ltd. The drug was administered via subcutaneous injection at a dosage of 0.2 ml (2 mg) once per week. Biochemical, body fat composition, and CGM parameters were assessed at baseline, Week 24, and Week 52 post-treatment.

### 2.3 Body composition assessment

Body composition was assessed using bioelectrical impedance analysis (InBody 770, Korea). During measurements, participants stood barefoot on the analyzer’s foot electrodes with proper positioning of forefeet and heels, while grasping hand electrodes with thumbs and fingers aligned. Participants fasted, emptied bladder/bowel, and rested at least 10 minutes in a quiet state before testing.

### 2.4 CGM assessment

**Figure 2:**
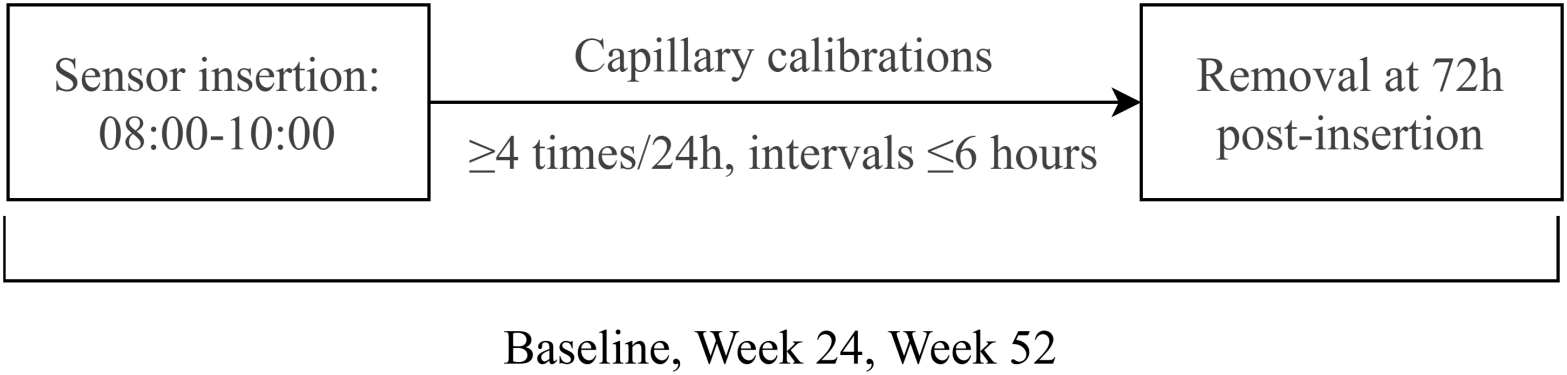
CGM flow chart.

This study implemented standardized CGM to evaluate glycemic fluctuations in subjects. All subjects underwent three 72-hour CGM sessions at baseline, Week 24, and Week 52 using the Medtronic CGMS-Gold system with Sof-sensors, administered by certified diabetes care specialists. The monitoring protocol specified sensor insertion between 08:00-10:00, with a minimum of four capillary blood glucose calibrations per 24-hour period, followed by sensor removal and data collection at 72 hours post-insertion (day 3, 08:00-10:00). Comprehensive glycemic variability analysis included calculation of: 24-hour mean blood glucose (MBG), glucose standard deviation (SD), coefficient of variation (CV), time in range (TIR), time above range (>10.0 mmol/L) and below range (<3.9 mmol/L), area under the curve for hyperglycemia (AUC>10.0 mmol/L), area under the curve for hypoglycemia (AUC<3.9 mmol/L), and documented hypoglycemic events. The CGM flow chart was described as **Figure 2**.

### 2.5 Statistical analysis

Data were analyzed by using SPSS 26.0. Normally distributed variables are expressed as mean ± SD (paired/independent t-tests). Non-normal variables are presented as median (interquartile range) (Wilcoxon signed-rank/Mann-Whitney U tests). *P*<0.05 was considered statistically significant.

## 3 Result

This study enrolled 78 patients with poorly controlled T2DM from Nanjing First Hospital. Two placebo-group subjects withdrew (one due to FBG >13.3 mmol/L at Week 8, one for personal reasons). During the trial, three participants (two from the Ebenatide group and one from the placebo group) missed CGM assessments due to equipment issues, while one additional participant (the Ebenatide group) failed to complete body composition analysis for personal reasons. In the overall analysis, all missing data from these participants were excluded. No other missing data were observed in the study. Baseline characteristics showed no statistically significant differences between the Ebenatide and placebo groups across all measured parameters (**Table 1**).

**Table 1:** Baseline characteristics of the patients.

Item	Total (N=76)	The Ebenatide Group (N=52)	The Placebo Group (N=24)	*P*
Sex (male/female)	55/21	37/15	18/6	0.789
Age (yrs.)	54.74 ± 10.01	54.92 ± 10.27	54.33 ± 9.63	0.675
SBP (mmHg)	131.63 ± 14.12	132.17 ± 13.34	130.46 ± 15.93	0.626
DBP (mmHg)	86.00 (81.75–90.00)	86.00 (83.00-90.00)	83.00 (80.50-87.00)	0.099
ALT (U/L)	28.00 (20.08-41.50)	28.00 (20.00-44.25)	27.50 (21.28-36.25)	0.817
AST (U/L)	19.50 (14.00-24.55)	22.00 (14.00-26.18)	18.50 (15.25-22.75)	0.648
TC (mmol/l)	4.81 ± 0.70	4.86 ± 0.69	4.68 ± 0.71	0.155
TG (mmol/l)	1.91 (1.38-2.74)	1.90 (1.32-2.74)	1.94 (1.44-2.92)	0.796
HDL (mmol/l)	1.18 ± 0.27	1.19 ± 0.29	1.15 ± 0.22	0.549
LDL (mmol/l)	2.87 ± 0.61	2.91 ± 0.63	2.77 ± 0.56	0.372
Cr (μmol/L)	65.37 ± 14.38	66.32 ± 15.73	63.31 ± 10.94	0.400
Hb (g/L)	150.92 ± 13.93	151.37 ± 11.94	149.96 ± 17.68	0.685
AMY (U/L)	59.37 ± 14.46	57.98 ± 13.87	62.33 ± 15.52	0.226
LIP (U/L)	86.00 (58.00-126.00)	82.00 (56.50-113.00)	105.00 (67.25-133.75)	0.380
FBG (mmol/l)	8.67 ± 1.62	8.59 ± 1.64	8.84 ± 1.60	0.601
FCP (ng/ml)	0.92 (0.75-1.17)	0.91 (0.75-1.14)	0.99 (0.74-1.23)	0.380
TyG	9.49 ± 0.56	9.46 ± 0.56	9.55 ± 0.56	0.523
HbA1c (%)	8.35 ± 1.05	8.48 ± 1.13	8.06 ± 0.76	0.188
Weight (kg)	74.53 ± 12.21	72.58 ± 9.94	78.27 ± 15.23	0.064
BMI (kg/m²)	26.50 ± 3.26	25.87 ± 2.70	27.90 ± 4.03	0.053
PBF (%)	30.23 ± 6.35	29.40 ± 6.11	32.06 ± 6.72	0.196
VFA (cm²)	103.11 ± 30.10	98.57 ± 25.27	113.24 ± 38.00	0.146
BFM (kg)	22.20 ± 6.21	21.06 ± 5.03	24.71 ± 7.88	0.067
SLM (kg)	47.85 ± 7.54	47.72 ± 6.33	48.15 ± 10.17	0.865
FFM (kg)	50.57 ± 7.93	50.44 ± 6.67	50.89 ± 10.69	0.865
SMM (kg)	27.97 ± 4.75	27.9 ± 4.00	28.15 ± 6.38	0.876
FMI (kg/m²)	8.14 ± 2.45	7.72 ± 2.25	9.06 ± 2.70	0.090
SMI (kg/m²)	7.60 (7.10-8.40)	7.50 (7.10-8.20)	8.10 (6.45-9.13)	0.249
WC (cm)	92.88 ± 8.46	92.49 ± 7.35	93.63 ± 10.38	0.599
HC (cm)	98.44 ± 5.13	97.51 ± 3.71	100.51 ± 7.10	0.069
WHtR	0.56 ± 0.05	0.55 ± 0.05	0.56 ± 0.05	0.711
MBG (mmol/l)	10.25 (8.77-12.30)	10.52 (8.54-12.32)	10.13 (9.24-11.26)	0.985
SD (mmol/l)	2.00 (1.54-2.44)	2.07 (1.70-2.39)	1.89 (1.48-2.51)	0.442
CV (%)	19.97 ± 5.55	20.40 ± 5.76	19.10 ± 5.10	0.363
TIR (%)	51.39 (17.10-76.04)	42.71 (16.84-77.26)	58.68 (39.41-72.92)	0.783
TBR (%)	0.00 (0.00-0.00)	0.00 (0.00-0.00)	0.00 (0.00-0.00)	0.319
TAR (%)	48.61 (23.96-82.90)	57.29 (22.74-83.16)	41.32 (27.08-60.59)	0.788
AUC<3.9 (mmol/L × h)	0.00 (0.00-0.00)	0.00 (0.00-0.00)	0.00 (0.00-0.00)	0.319
AUC>10 (mmol/L × h)	1383.75 (384.25-3567.25)	1554.50 (362.50-3904.00)	1157.00 (391.50-2617.50)	0.712

∗*P*<0.05, ∗∗*P*<0.01; SP (mmHg), systolic pressure; DP (mmHg), diastolic pressure; ALT (U/L), alanine aminotransferase; AST (U/L), aspartate aminotransferase; TC (mmol/l), total cholesterol; TG (mmol/l), triglycerides; HDL (mmol/l), high-density lipoprotein; LDL (mmol/l), low-density lipoprotein; Cr (μmol/L), creatinine; Hb (g/L), hemoglobin; AMY (U/L), amylase; LIP (U/L), lipase; FBG (mmol/l), fasting blood glucose; FCP (ng/ml), fasting C-peptide; TyG, triglyceride-glucose index; HbA1c (%), glycated hemoglobin A1c; BMI (kg/m²), body mass index; PBF (%), percent body fat; VFA (cm²), visceral fat area; BFM (kg), body fat mass; SLM (kg), skeletal lean mass; FFM (kg), fat free mass; SMM (kg), skeletal muscle mass; FMI (kg/m²), fat mass index; SMI (kg/m²), skeletal muscle mass index; WC (cm), waist circumference; HC (cm), hip circumference; WHtR, waist-to-height ratio; MBG (mmol/l), mean blood glucose; SD (mmol/l), standard deviation; CV (%), coefficient of variation; TIR (%), percentage of time with glucose levels within 3.9-10.0 mmol/L (target range); TBR (%), percentage of time <3.9 mmol/L; TAR (%), percentage of time >10.0 mmol/L; AUC<3.9 (mmol/L × h), the incremental area under curve of glucose<3.9mmol/L; AUC>10 (mmol/L × h), the incremental area under curve of glucose>10.0mmol/L.

### 3.1 Effects of Ebenatide on glycemic metabolism

**Figure 3:**
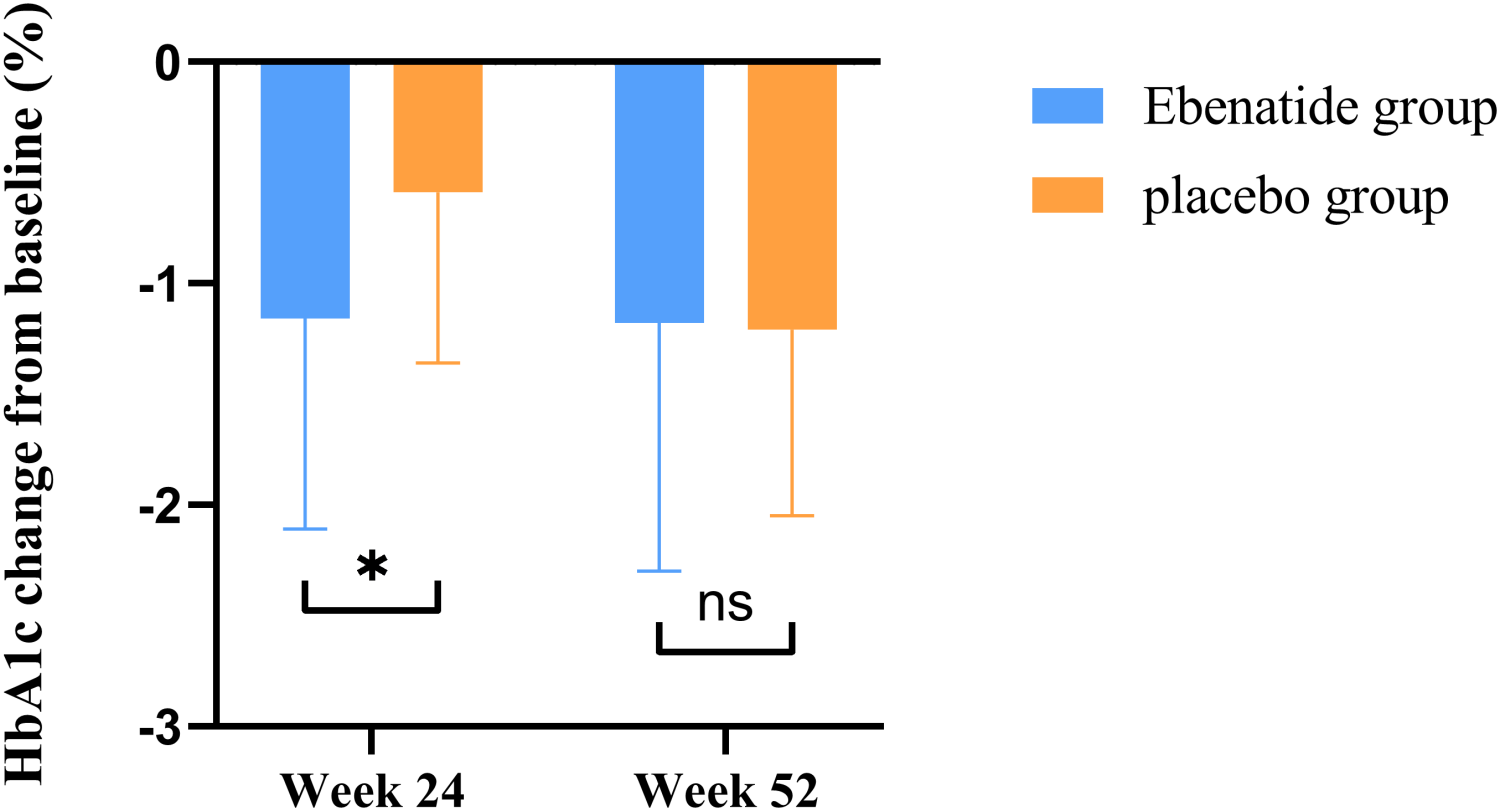
Changes in HbA1c from baseline in the Ebenatide group versus placebo group after treatment. Comparison of the magnitude of HbA1c reduction from baseline between the Ebenatide and placebo groups. *P<0.05; ns, not statistically significant between groups.

In the Ebenatide group, significant reductions were observed at Week 24 compared to baseline in both FBG (8.59 ± 1.64 vs. 7.94 ± 1.50 mmol/L, *P* = 0.025) and HbA1c (8.48 ± 1.13% vs. 7.32 ± 0.89%, *P* < 0.001). Meanwhile, the placebo group also demonstrated improvement in glycemic control, with a decrease in HbA1c (8.06 ± 0.76% vs. 7.47 ± 0.69%, *P* = 0.002). The reduction in HbA1c was significantly greater in the Exenatide group compared to the placebo group (mean difference = 0.57% [95% CI: 0.14 to 1.04], *P* = 0.011) (**Figure 3**).

After 52 weeks of treatment, the Ebenatide group maintained sustained reductions in HbA1c compared to baseline (8.48 ± 1.13% vs. 7.30 ± 0.91%, *P* < 0.001), the reduction magnitude plateaued after Week 24. The placebo group similarly showed significant HbA1c decreases at Week 52 versus both baseline and Week 24 measurements (baseline: 8.06 ± 0.76% vs. 24 weeks: 7.47 ± 0.69% vs. 52 weeks: 6.85 ± 0.67%; *P* < 0.001). The placebo group demonstrated more pronounced reductions in both FBG and HbA1c from Week 24 compared to the Ebenatide group during the same period (all *P* < 0.05).

Using HbA1c <7% as the glycemic target criterion, significant differences in achievement rates were observed between the Ebenatide and placebo groups. At Week 24, the target achievement rate in the Ebenatide group was significantly higher than that in the placebo group (37% vs. 17%). By Week 52, the Ebenatide group maintained its achievement rate at 37%, while the placebo group showed a marked increase to 58% (**Table 2**).

**Table 2:** HbA1c target achievement in the Ebenatide group versus placebo group (n=52).

	The Ebenatide Group (N=52)	The Placebo Group (N=24)
Week 24 Achievement Rate (%)	37	17
Week 52 Achievement Rate (%)	37	58
Percentage Change From Week 24 to Week 52(%)	0	41

HbA1c <7% was defined as achieving the HbA1c target.

By Week 24, the Ebenatide group exhibited a reduction in TyG compared to baseline, though this change did not reach statistical significance. At Week 52, TyG showed significant decreases relative to both baseline and the 24th week in the Ebenatide group (baseline: 9.46 ± 0.56 vs. 24 weeks: 9.28 ± 0.60 vs. 52 weeks: 8.76 ± 0.62; *P* < 0.001). Similarly, in the placebo group at the 52nd week, TyG demonstrated significant reductions versus baseline and the 24th week measurements (baseline: 9.55 ± 0.56 vs. 24 weeks: 9.33 ± 0.47 vs. 52 weeks: 8.69 ± 0.43; *P* < 0.001). Detailed results are shown in **Table 3**.

**Table 3:** Comparison of glucose metabolism parameters before and after treatment in the Ebenatide group (n=52).

Item	Baseline	Week 24	Week 52	*P*1	*P*2	*P*3
FBG (mmol/l)	8.59 ± 1.64	7.94 ± 1.50	8.36 ± 1.66	0.025*	0.640	0.038*
FCP (ng/ml)	0.91 (0.75-1.14)	0.86 (0.75-1.09)	0.94 (0.75-1.14)	0.143	0.878	0.179
TyG	9.46 ± 0.56	9.28 ± 0.60	8.76 ± 0.62	0.063	<0.001**	<0.001**
HbA1c (%)	8.48 ± 1.13	7.32 ± 0.89	7.30 ± 0.91	<0.001**	<0.001**	0.458

*P*1: baseline vs. Week 24, *P*2: baseline vs. Week 52, *P*3: Week 24 vs. Week 52; ∗*P*<0.05, ∗∗*P*<0.01; FBG (mmol/l), fasting blood glucose; FCP (ng/ml), fasting C-peptide; TyG, triglyceride-glucose index; HbA1c (%), glycated hemoglobin A1c.

### 3.2 Effects of Ebenatide on CGM parameters

After 24 weeks of treatment, the Ebenatide group exhibited a significant increase in TIR compared to baseline (42.71% [16.84-77.26] vs. 81.94% [63.19-97.92], *P* < 0.001). In contrast, while the placebo group demonstrated improvement in TIR, change did not reach statistical significance. The Ebenatide group showed superior improvement in TIR relative to the placebo group (mean difference = -16.92% [95% CI: -32.27 to -1.57], *P* = 0.031).

By Week 52, the Exenatide group maintained higher TIR versus baseline (42.71% [16.84-77.26] vs. 91.67% [77.43-97.21], *P <*0.001), with no significant additional gain after Week 24. The placebo group demonstrated significant TIR improvements at Week 52 versus both baseline and Week 24 measurements (baseline: 58.68% [39.41-72.92] vs. 24 weeks: 70.49% [60.76-83.33] vs. 52 weeks: 98.61% [81.94-100.00]; *P* < 0.05). However, no statistically significant between-group differences in TIR were observed at Week 52.

After 24 weeks of treatment, the Ebenatide group showed significant reductions from baseline in MBG, SD, TAR, and AUC >10 (all *P* < 0.05). In contrast, the placebo group demonstrated improvement only in MBG (*P* < 0.05) without significant changes in other metrics. Comparative analysis revealed that the improvement in TAR was significantly greater in the Ebenatide group than in the placebo group (*P* = 0.024). After 52 weeks, the Ebenatide group exhibited significant reductions from baseline in MBG, SD, TAR, and AUC>10 (all *P* < 0.001), with no statistically significant differences compared to Week 24 values. The placebo group showed significant improvements in MBG and TAR versus both baseline and Week 24 measurements (both *P* < 0.01). However, no significant between-group differences were observed for other CGM parameters at Week 52.

Throughout the treatment period, both the Ebenatide and placebo groups maintained an undetectable TBR and AUC<3.9.

**Figure 4:**
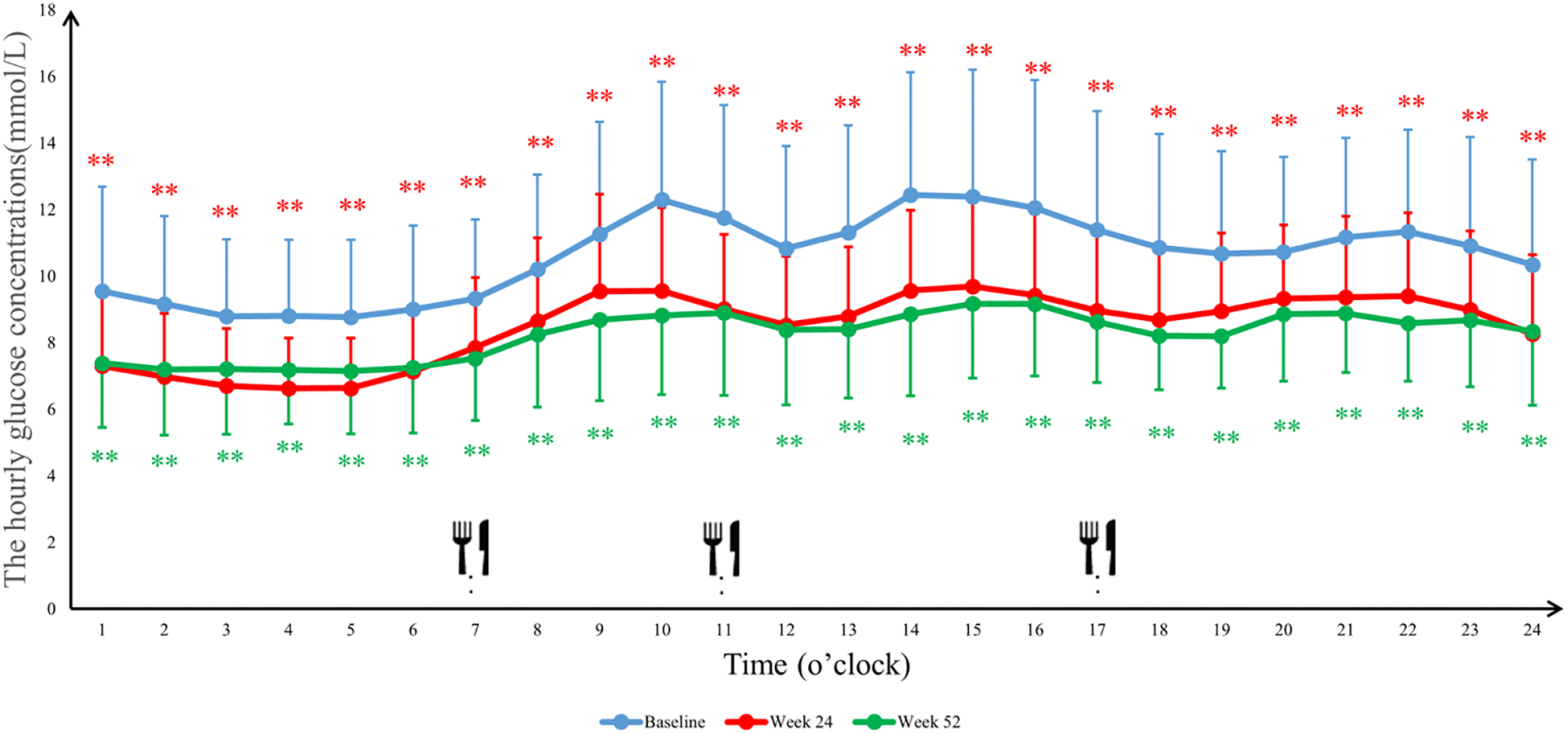
The average blood glucose concentrations per hour in the Ebenatide group. 24-h continuous glucose monitoring (CGM) profiles of the Ebenatide group at baseline, Week 24, and Week 52. Data are presented as mean ± SD. Meal times were 07:00, 11:00, and 17:00 for breakfast, lunch, and dinner. ∗*P*<0.05, ∗∗*P*<0.01.

**Figure 5:**
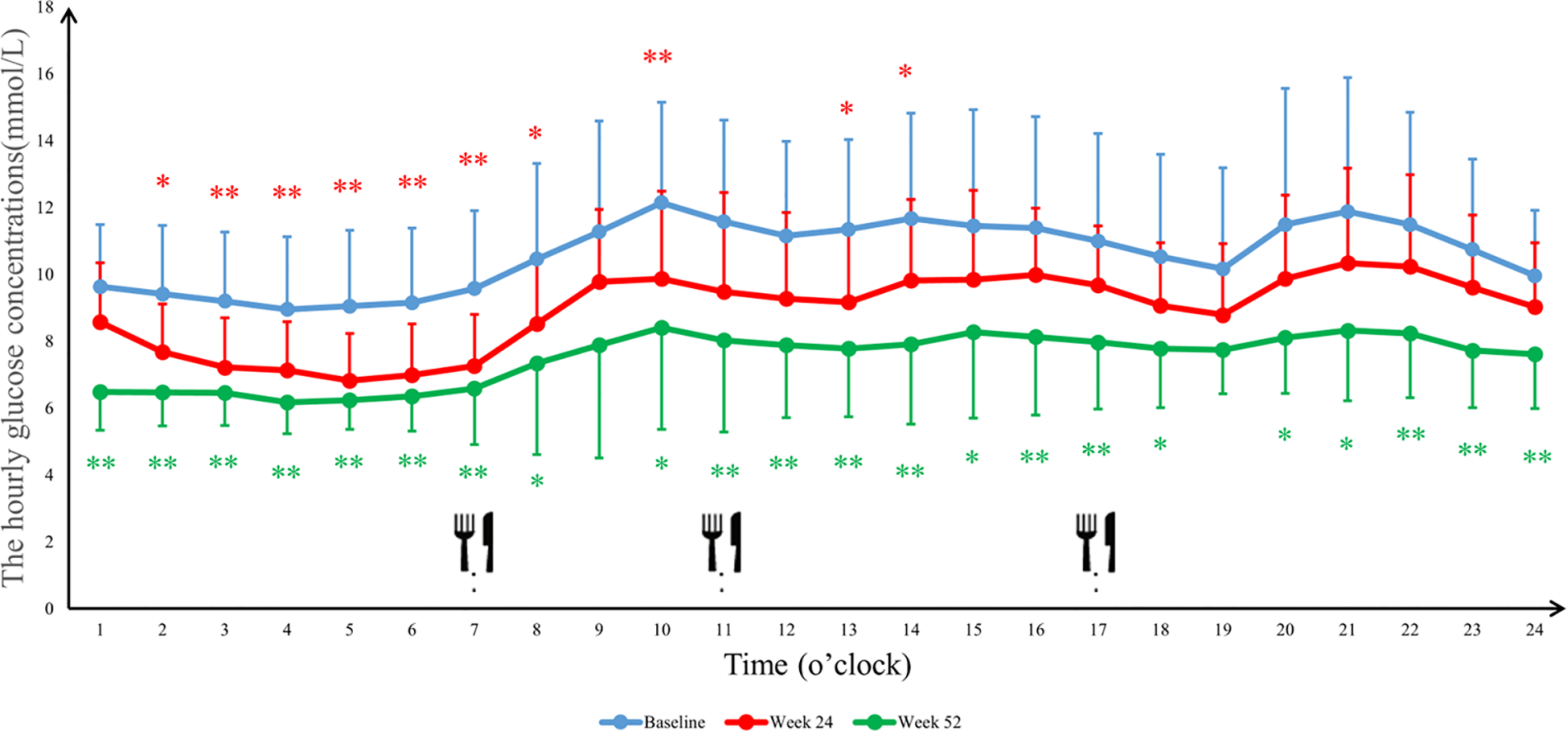
The average blood glucose concentrations per hour in the placebo group. 24-h continuous glucose monitoring (CGM) profiles of the placebo group at baseline, Week 24, and Week 52. Data are presented as mean ± SD. Meal times were 07:00, 11:00, and 17:00 for breakfast, lunch, and dinner. ∗*P*<0.05, ∗∗*P*<0.01.

The 24-hour glucose profiles demonstrated that the Ebenatide group achieved significant reductions in hourly glucose levels at both Week 24 and Week 52 compared to baseline (all *P* < 0.01), with particularly pronounced decreases during postprandial periods. In the placebo group, glucose-lowering effects were more marked at Week 52 than Week 24, with statistically significant reductions observed specifically during post-lunch (11:00-13:00, *P* < 0.01) and post-dinner (20:00-24:00, *P* < 0.05) intervals (**Figures 4**, **5**). Detailed results are shown in **Table 4**.

**Table 4:** Comparison of CGM parameters before and after treatment in the Ebenatide group (n=50).

Item	Baseline	Week 24	Week 52	*P*1	*P*2	*P*3
MBG (mmol/l)	10.52 (8.54-12.32)	8.21 (7.49-9.42)	8.17 (7.35-8.75)	<0.001**	<0.001**	0.149
SD (mmol/l)	2.07 (1.70-2.39)	1.77 (1.10-2.40)	1.44 (1.22-1.91)	0.029*	<0.001**	0.055
CV (%)	20.40 ± 5.76	21.31 ± 8.39	18.22 ± 4.92	0.861	0.086	0.102
TIR (%)	42.71 (16.84-77.26)	81.94 (63.19-97.92)	91.67 (77.43-97.21)	<0.001**	<0.001**	0.119
TBR (%)	0.00 (0.00-0.00)	0.00 (0.00-0.00)	0.00 (0.00-0.00)	0.173	0.655	0.080
TAR (%)	57.29 (22.74-83.16)	14.93 (2.08-36.63)	8.33 (2.79-22.57)	<0.001**	<0.001**	0.149
AUC<3.9 (mmol/L × h)	0.00 (0.00-0.00)	0.00 (0.00-0.00)	0.00 (0.00-0.00)	0.173	0.655	0.080
AUC>10 (mmol/L × h)	1554.50 (362.50-3904.00)	378.00 (8.50-1188.50)	116.25 (12.38-540.13)	<0.001**	<0.001**	0.115

*P*1: baseline vs. Week 24, *P*2: baseline vs. Week 52, *P*3: Week 24 vs. Week 52; ∗*P*<0.05, ∗∗*P*<0.01; MBG (mmol/l), mean blood glucose; SD (mmol/l), standard deviation; CV (%), coefficient of variation; TIR (%), percentage of time with glucose levels within 3.9-10.0 mmol/L (target range); TBR (%), percentage of time <3.9 mmol/L; TAR (%), percentage of time >10.0 mmol/L; AUC<3.9 (mmol/L × h), the incremental area under curve of glucose<3.9mmol/L; AUC>10 (mmol/L × h), the incremental area under curve of glucose>10.0mmol/L.

### 3.3 Effects of Ebenatide on body composition parameters

After 24 weeks of treatment, the Ebenatide group exhibited significant reductions from baseline in body weight, BMI, percent body fat (PBF), visceral fat area (VFA), body fat mass (BFM), fat mass index (FMI), WC, and WHtR (all *P* < 0.05). No significant changes from baseline were observed in skeletal lean mass (SLM), fat free mass (FFM), skeletal muscle mass (SMM), or skeletal muscle mass index (SMI) in either the Ebenatide or placebo groups, and no statistically significant differences between the two groups.

After 52 weeks of treatment, the Ebenatide group maintained significant reductions from baseline in body weight, WC, and WHtR (all *P* < 0.05). However, no statistically significant differences were observed for BMI, PBF, VFA, BFM, or FMI compared to baseline, though BMI continued to show a downward trend, while PBF, VFA, BFM, and FMI exhibited mild increases relative to Week 24 measurements. Between Week 24 and Week 52, the Ebenatide group demonstrated significant decreases in FFM, SMM, and SMI (all *P* < 0.05), though these parameters did not differ significantly from baseline levels. Similarly, the placebo group also showed significant reductions in SLM, FFM, and SMM (all *P* < 0.05). Detailed results are shown in **Table 5**.

**Table 5:** Comparison of body composition parameters before and after treatment in the Ebenatide group (n=51).

Item	Baseline	Week 24	Week 52	*P*1	*P*2	*P*3
Weight (kg)	72.58 ± 9.94	71.52 ± 10.07	70.72 ± 9.48	0.007**	0.002**	0.070
BMI (kg/m²)	25.87 ± 2.70	25.56 ± 2.58	25.42 ± 2.57	0.026*	0.064	0.478
PBF (%)	29.40 ± 6.11	28.00 ± 6.63	29.09 ± 6.77	0.003**	0.596	0.053
VFA (cm²)	98.57 ± 25.27	91.89 ± 25.63	93.57 ± 25.55	0.005**	0.055	0.487
BFM (kg)	21.06 ± 5.03	19.87 ± 5.33	20.53 ± 5.62	0.005**	0.352	0.199
SLM (kg)	47.72 ± 6.33	48.12 ± 6.57	47.43 ± 6.81	0.134	0.485	0.115
FFM (kg)	50.44 ± 6.67	50.89 ± 6.90	49.85 ± 7.07	0.107	0.045*	0.001**
SMM (kg)	27.90 ± 4.00	28.13 ± 4.17	27.51 ± 4.22	0.161	0.027*	0.001**
FMI (kg/m²)	7.72 ± 2.25	7.28 ± 2.33	7.50 ± 2.41	0.007**	0.321	0.243
SMI (kg/m²)	7.50 (7.10-8.20)	7.60 (7.00-8.20)	7.60 (6.90-8.20)	0.479	0.047*	0.044*
WC (cm)	92.49 ± 7.35	90.18 ± 7.18	90.00 ± 6.79	<0.001**	<0.001**	0.649
HC (cm)	97.51 ± 3.71	97.12 ± 3.70	97.02 ± 3.73	0.074	0.197	0.716
WHtR	0.55 ± 0.05	0.54 ± 0.05	0.54 ± 0.05	<0.001**	0.018*	0.633

*P*1: baseline vs. Week 24, *P*2: baseline vs. Week 52, *P*3: Week 24 vs. Week 52; ∗*P*<0.05, ∗∗*P*<0.01; BMI (kg/m²), body mass index; PBF (%), percent body fat; VFA (cm²), visceral fat area; BFM (kg), body fat mass; SLM (kg), skeletal lean mass; FFM (kg), fat free mass; SMM (kg), skeletal muscle mass; FMI (kg/m²), fat mass index; SMI (kg/m²), skeletal muscle mass index; WC (cm), waist circumference; HC (cm), hip circumference; WHtR, waist-to-height ratio.

### 3.4 Effects of Ebenatide on conventional metabolic parameters

After 24 weeks of treatment, the Ebenatide group demonstrated significant reductions from baseline in SBP, DBP, and hemoglobin levels, along with significant increases in serum amylase and lipase levels (all *P* < 0.05). These trends in blood pressure and hemoglobin persisted after 52 weeks (all *P* < 0.05 versus baseline), while the elevations in serum amylase and lipase remained significantly higher than baseline at both time points (both *P* < 0.05). At Week 52, the placebo group showed reduced SBP, DBP and TC, but increased serum lipase and amylase versus baseline. Compared to Week 24, further decreases were seen in SBP and TC, while only serum amylase increased. All these changes reached statistical significance (all *P* < 0.05). Detailed results are shown in **Table 6**.

**Table 6:** Comparison of conventional metabolic parameters before and after treatment in the Ebenatide group (n=52).

Item	Baseline	Week 24	Week 52	*P*1	*P*2	*P*3
SBP (mmHg)	132.17 ± 13.34	125.31 ± 10.58	124.90 ± 14.96	0.001**	0.004**	0.802
DBP (mmHg)	86.00 (83.00-90.00)	82.00 (76.00-86.50)	82.00 (74.00-86.00)	<0.001**	<0.001**	0.501
ALT (U/L)	28.00 (20.00-44.25)	23.10 (15.60-32.10)	25.85 (18.25-37.78)	0.015*	0.345	0.259
AST (U/L)	22.00 (14.00-26.18)	18.20 (14.00-21.50)	19.40 (14.88-26.20)	0.198	0.827	0.294
TC (mmol/l)	4.86 ± 0.69	4.81 ± 0.77	4.70 ± 0.90	0.893	0.144	0.181
TG (mmol/l)	1.90 (1.32-2.74)	1.76 (1.13-2.50)	1.90 (1.46-2.47)	0.373	0.397	0.433
HDL (mmol/l)	1.19 ± 0.29	1.23 ± 0.31	1.20 ± 0.32	0.392	0.980	0.122
LDL (mmol/l)	2.91 ± 0.63	2.91 ± 0.68	2.88 ± 0.69	0.963	0.481	0.443
Cr (μmol/L)	66.32 ± 15.73	68.03 ± 17.96	66.28 ± 15.60	0.168	0.923	0.025*
Hb (g/L)	151.37 ± 11.94	145.92 ± 12.29	143.72 ± 12.20	<0.001**	<0.001**	0.017*
AMY (U/L)	57.98 ± 13.87	67.79 ± 17.17	69.00 ± 16.17	<0.001**	<0.001**	0.579
LIP (U/L)	82.00 (56.50-113.00)	95.50 (70.50-148.75)	107.00 (74.00-159.00)	<0.001**	<0.001**	0.981

*P*1: baseline vs. Week 24, *P*2: baseline vs. Week 52, *P*3: Week 24 vs. Week 52; ∗*P*<0.05, ∗∗*P*<0.01; SP (mmHg), systolic pressure; DP (mmHg), diastolic pressure; ALT (U/L), alanine aminotransferase; AST (U/L), aspartate aminotransferase; TC (mmol/l), total cholesterol; TG (mmol/l), triglycerides; HDL (mmol/l), high-density lipoprotein; LDL (mmol/l), low-density lipoprotein; Cr (μmol/L), creatinine; Hb (g/L), hemoglobin; AMY (U/L), amylase; LIP (U/L), lipase.

## 4 Discussion

This study demonstrated that Ebenatide showed significant and sustained glucose-lowering effects. Although no further reduction in HbA1c was observed between Week 24 and Week 52, CGM revealed further improvement in TIR, indicating that long-term Ebenatide treatment could progressively enhance TIR. The CGM results demonstrated that Ebenatide treatment reduced glycemic variability, with hourly glucose declines particularly prominent in postprandial glucose. Previous studies have shown that long-term glycemic variability is strongly associated with an increased risk of stroke and cardiovascular events in T2DM patients (16, 17). Iatrogenic hypoglycemia may contribute to emotional burden (18), and is linked to falls and cognitive impairment, while hypoglycemia in T2DM patients is associated with higher cardiovascular event rates (19). No hypoglycemic episodes occurred during Ebenatide treatment.

In recent years, TyG has gained increasing recognition for its role in assessing insulin resistance and predicting glycemic control in T2DM. Elevated TyG levels have been consistently associated with higher risks of diabetic kidney disease, coronary artery disease, heart failure, atrial fibrillation, and ischemic stroke (12, 13, 20, 21). The present study demonstrates that Ebenatide treatment produces sustained reductions in TyG, with progressively enhanced effects over time. Notably, statistically significant differences in TyG were observed between baseline and Week 52, indicating that Ebenatide’s beneficial effects on insulin resistance intensify with prolonged administration. These findings suggest that extended Ebenatide treatment could provide durable improvements in insulin resistance, though longer-term studies are needed to fully characterize these sustained metabolic benefits.

GLP-1 RAs demonstrate heterogeneous glycemic efficacy across clinical trials. In our study, Ebenatide achieved a mean HbA1c reduction of 1.18% at Week 52. For context, other GLP-1RAs reported the following HbA1c changes in separate trials: Exenatide (Δ -0.81%) (22), Polyethylene Glycol Loxenatide (Δ -1.01%) (23), Semaglutide (Δ -1.09% to -1.59%) (24), and Tirzepatide (Δ -1.60% to -1.96%) (24).

Numerous studies have demonstrated that Tirzepatide exhibits superior weight-loss efficacy compared to Semaglutide (25), with optimal performance in reducing both BMI and waist circumference (19). Although Ebenatide’s weight-loss effect is not as pronounced as above two agents, it shows significant advantages over Loxenatide (22, 26), which is a homologue of Ebenatide with inferior weight-loss outcomes. Body composition analysis revealed that Ebenatide-treated patients exhibited marked reductions in adiposity metrics as early as 24 weeks of treatment. These findings not only corroborate Ebenatide’s effectiveness in weight management but also highlight its positive role in improving fat distribution, particularly in reducing visceral adipose tissue. Excessive adipose tissue, especially visceral adipose tissue, exacerbates insulin resistance and accelerates T2DM progression (27). Evidence indicates positive correlations between HbA1c/FBG and obesity-related indices (28), suggesting that Ebenatide may confer dual benefits via direct glucose-lowering and indirect adiposity-modifying mechanisms. However, after 52 weeks, minor rebounds in some adiposity parameters were observed, potentially reflecting the development of tolerance with prolonged Ebenatide use. The phenomenon consistent with prior reports of tolerance induction during long-term therapy with other extended-action GLP-1RAs (29).

Diabetes and sarcopenia mutually exacerbate each other thus forming a vicious cycle. The reduction in muscle mass increases the risk of various diabetic complications, including diabetic nephropathy and peripheral neuropathy (30–32). We observed preserved muscle-related parameters (FFM, SMM, and SMI) after 24 weeks of treatment, while these indices demonstrated decline at Week 52. These findings suggest that short-term Ebenatide treatment (≤24 weeks) has minimal impact on muscle mass, whereas prolonged administration (52 weeks) may exert potentially adverse effects on muscular composition. This aligns with a previous study demonstrating that Liraglutide treatment reduced both lean body mass and adiposity in patients (33). However, another study found that Semaglutide preserved muscle mass to some extent while reducing fat mass (34). Weight loss surgery is frequently associated with reductions in lean body mass, FFM, and SMM (35). In the present study, the observed decline in muscle mass may similarly stem from energy metabolism dysregulation secondary to appetite suppression and reduced caloric intake. The detailed mechanisms underlying these changes in muscle-related parameters require further investigation.

Our study demonstrated significant reductions in both systolic and diastolic blood pressure from baseline at Week 24 and Week 52 with Ebenatide treatment. Studies have identified coefficients of variation for SBP, DBP, FBG, and HbA1c as important predictors of stroke, cardiovascular events, and related mortality (36). The concurrent blood pressure-lowering and glucose-lowering effects of Ebenatide suggest its potential cardioprotective benefits through multiple mechanisms.

Additionally, Ebenatide treatment exhibited measurable effects on serum amylase elevation, lipase elevation, and hemoglobin reduction. Although no cases of pancreatitis or anemia diagnosed, considered pancreatitis has been frequently reported as a GLP-1RAs-associated adverse drug reaction (37), regular monitoring remains essential to ensure treatment safety. The observed hemoglobin reduction represents an uncommon finding among GLP-1RAs, warranting further investigation into Ebenatide-specific mechanisms affecting hemoglobin levels.

Studies have identified gastrointestinal reactions as frequent adverse effects of GLP-1RAs treatment, with nausea, vomiting, diarrhea, and constipation being the most commonly reported (38). During the study, patients in Ebenatide group experienced mild-to-moderate gastrointestinal reactions in the first 24 weeks of treatment, primarily self-limiting nausea (9.6%) and vomiting (5.8%), which is consistent with the GLP-1 receptor agonist class. These symptoms showed gradual amelioration with prolonged treatment duration. Further studies with larger sample sizes are required to better characterize the gastrointestinal tolerability profile of Ebenatide.

Collectively, Ebenatide demonstrates favorable glycemic control and weight reduction effects among GLP-1 RAs, with additional blood pressure-lowering benefits not consistently observed with other agents in this class. Its safety profile is characterized by manageable gastrointestinal adverse events, consistent with the established tolerability profile of GLP-1-based therapies. However, these numerical differences must be interpreted with caution due to variations in trial designs, and the relative efficacy ranking remains to be validated in head-to-head trials.

The placebo group also exhibited improved glycemic control and reductions in body weight and fat mass prior to drug administration, which may be attributed to strict dietary and exercise interventions. As a guideline-recommended treatment strategy, the efficacy of lifestyle modifications (including dietary control and physical activity) in diabetes management has been well documented in multiple clinical studies (39, 40).

Furthermore, we separately analyzed data from the Ebenatide monotherapy group and the Ebenatide+metformin group. The overall findings were consistent with the total population results. Due to its smaller sample size, certain outcomes in the Ebenatide+metformin group demonstrated only directional trends without reaching statistical significance. These observations warrant further investigation with larger-scale clinical trials for definitive evaluation.

## 5 Conclusion

Ebenatide treatment for 24 weeks significantly improved multiple metabolic parameters including HbA1c, TIR, TyG, weight, WC, and WHtR. These therapeutic benefits were sustained for at least 52 weeks. However, close monitoring of serum amylase, lipase, hemoglobin levels, and muscle mass is recommended during treatment.

## Data Availability

The original contributions presented in the study are included in the article/supplementary material, further inquiries can be directed to the corresponding authors.
