# Supplementary material for: Effect of Ebenatide on glycemic metabolism and body fat in patients with type 2 diabetes mellitus
Source: Front Endocrinol (Lausanne). 2025 Jun 18;16:1622526. doi: 10.3389/fendo.2025.1622526 (PMC12213378; doi:10.3389/fendo.2025.1622526)
Supplement: Supplementary file 1 [file Table1.docx]

Supplementary figure 1: Comparison before and after treatment in the placebo group

|  | Baseline | Week 24 | Week 52 | *P*1 | *P*2 | *P*3 |
| --- | --- | --- | --- | --- | --- | --- |
| SBP(mmHg) | 130.46 ± 15.93 | 128.87 ± 11.83 | 122.00 ± 11.48 | 0.332 | 0.005** | 0.003** |
| DBP (mmHg) | 83.00（80.50-87.00） | 81.50（78.00-84.00） | 81.00（76.50-84.50) | 0.325 | 0.029* | 0.057 |
| ALT(U/L) | 27.50（21.28-36.25） | 23.05（18.98-28.63） | 26.25（18.05-37.00) | 0.025* | 0.145 | 0.179 |
| AST(U/L) | 18.50（15.25-22.75） | 19.80（15.60-21.48） | 18.55（15.88-23.28) | 0.909 | 0.989 | 0.909 |
| TC(mmol/l) | 4.68 ± 0.71 | 4.70 ± 0.61 | 4.20 ± 0.81 | 0.444 | 0.009** | 0.004** |
| TG(mmol/l) | 1.94（1.44-2.92） | 1.45（1.19-2.63） | 1.73（1.22-2.23） | 0.123 | 0.057 | 0.530 |
| HDL(mmol/l) | 1.15 ± 0.22 | 1.25 ± 0.25 | 1.18 ± 0.19 | 0.055 | 0.458 | 0.074 |
| LDL(mmol/l) | 2.77 ± 0.56 | 2.88 ± 0.54 | 2.64 ± 0.78 | 0.214 | 0.304 | 0.114 |
| Cr (μmol/L) | 63.31 ± 10.94 | 65.63 ± 13.37 | 66.08 ± 13.64 | 0.053 | 0.052 | 0.703 |
| Hb(g/L) | 149.96 ± 17.68 | 149.04 ± 16.71 | 146.08 ± 19.12 | 0.640 | 0.071 | 0.046 |
| AMY(U/L) | 62.33 ± 15.52 | 70.25 ± 19.74 | 79.00 ± 19.09 | 0.001** | ＜0.001** | 0.010* |
| LIP (U/L) | 105.00（67.25-133.75） | 111.00（62.00-150.25） | 156.00（92.00-188.00） | 0.338 | ＜0.001** | 0.005 |
| FBG(mmol/l) | 8.84 ± 1.60 | 8.40 ± 1.07 | 8.12 ± 1.52 | 0.236 | 0.032* | 0.378 |
| FCP(ng/ml) | 0.99（0.74-1.23） | 0.99（0.74-1.32） | 1.07（0.69-1.31） | 0.644 | 0.297 | 0.178 |
| TyG | 9.55 ± 0.56 | 9.33 ± 0.47 | 8.69 ± 0.43 | 0.133 | ＜0.001** | ＜0.001** |
| HbA1c(%) | 8.06 ± 0.76 | 7.47 ± 0.69 | 6.85 ± 0.67 | 0.002** | ＜0.001** | ＜0.001** |
| Weight (kg) | 78.27 ± 15.23 | 77.69 ± 15.48 | 76.44 ± 15.64 | 0.139 | 0.002** | 0.008** |
| BMI(kg/m²) | 27.90 ± 4.03 | 27.46 ± 4.02 | 27.24 ± 4.24 | 0.014* | 0.012* | 0.390 |
| PBF(%) | 32.06 ± 6.72 | 31.01 ± 5.84 | 31.25 ± 6.17 | 0.025* | 0.222 | 0.730 |
| VFA(cm²) | 113.24 ± 38.00 | 106.44 ± 32.18 | 106.47 ± 34.52 | 0.014* | 0.082 | 0.992 |
| BFM(kg) | 24.71 ± 7.88 | 23.66 ± 6.95 | 23.70 ± 7.61 | 0.026* | 0.107 | 0.956 |
| SLM(kg) | 48.15 ± 10.17 | 48.28 ± 9.91 | 47.62 ± 10.25 | 0.769 | 0.158 | 0.038* |
| FFM(kg) | 50.89 ± 10.69 | 51.07 ± 10.41 | 50.38 ± 10.75 | 0.685 | 0.175 | 0.032* |
| SMM(kg) | 28.15 ± 6.38 | 28.22 ± 6.27 | 27.77 ± 6.42 | 0.780 | 0.091 | 0.028* |
| FMI(kg/m²) | 9.06 ± 2.70 | 8.61 ± 2.37 | 8.61 ± 2.52 | 0.010* | 0.062 | 1.000 |
| SMI(kg/m²) | 8.10（6.45-9.13） | 7.90（6.45-8.90） | 7.65（6.18-9.10） | 0.171 | 0.053 | 0.139 |
| WC(cm) | 93.63 ± 10.38 | 92.25 ± 10.04 | 91.76 ± 10.69 | 0.002** | 0.010* | 0.282 |
| HC(cm) | 100.51 ± 7.10 | 100.03 ± 6.91 | 99.74 ± 7.47 | 0.043* | 0.043* | 0.522 |
| WHtR | 0.56 ± 0.05 | 0.55 ± 0.04 | 0.55 ± 0.05 | 0.007** | 0.066 | 0.751 |
| MBG(mmol/l) | 10.13（9.24-11.26） | 8.86（7.96-9.88） | 6.91（6.36-8.38） | 0.014* | 0.004** | 0.023* |
| SD(mmol/l) | 1.89（1.48-2.51） | 1.89（1.47-2.27） | 1.40（0.94-1.67） | 0.900 | 0.122 | 0.144 |
| CV(%) | 19.10 ± 5.10 | 21.71 ± 7.50 | 17.67 ± 6.52 | 0.223 | 0.709 | 0.285 |
| TIR(%) | 58.68（39.41-72.92） | 70.49（60.76-83.33） | 98.61（81.94-100.00） | 0.078 | 0.006** | 0.016* |
| TBR(%) | 0.00（0.00-0.00） | 0.00（0.00-0.00） | 0.00（0.00-0.00） | 0.317 | 0.317 | 0.317 |
| TAR(%) | 41.32（27.08-60.59) | 29.51（16.67-39.24） | 1.39（0.00-18.06) | 0.077 | 0.005** | 0.016* |
| AUC＜3.9  (mmol/L × h) | 0.00（0.00-0.00） | 0.00（0.00-0.00） | 0.00（0.00-0.00） | 0.317 | 0.317 | 0.317 |
| AUC＞10  (mmol/L × h) | 1157.00（391.50-2617.50） | 590.00（303.00-1135.00） | 5.50（0.00-311.25） | 0.084 | 0.028 | 0.091 |

*P*1: baseline vs. Week 24, *P*2: baseline vs. Week 52, *P*3: Week 24 vs. Week 52; ∗*P*<0.05, ∗∗*P*<0.01; SP (mmHg): systolic pressure; DP (mmHg): diastolic pressure; ALT (U/L): alanine aminotransferase; AST (U/L): aspartate aminotransferase; TC (mmol/l): total cholesterol; TG (mmol/l): triglycerides; HDL (mmol/l): high-density lipoprotein; LDL (mmol/l): low-density lipoprotein; Cr (μmol/L): creatinine; Hb (g/L): hemoglobin; AMY (U/L): amylase; LIP (U/L): lipase; FBG (mmol/l): fasting blood glucose; FCP (ng/ml): fasting C-peptide; TyG: triglyceride-glucose index; HbA1c (%): glycated hemoglobin A1c; BMI (kg/m²): body mass index; PBF (%): percent body fat; VFA (cm²): visceral fat area; BFM (kg): body fat mass; SLM (kg): skeletal lean mass; FFM (kg): fat free mass; SMM (kg): skeletal muscle mass; FMI (kg/m²): fat mass index; SMI (kg/m²): skeletal muscle mass index; WC (cm): waist circumference; HC (cm): hip circumference; WHtR: waist-to-height ratio; MBG (mmol/l): mean blood glucose; SD (mmol/l): standard deviation; CV (%): coefficient of variation; TIR (%): percentage of time with glucose levels within 3.9-10.0 mmol/L (target range); TBR (%): percentage of time <3.9 mmol/L; TAR (%): percentage of time >10.0 mmol/L; AUC＜3.9 (mmol/L × h): the incremental area under curve of glucose＜3.9mmol/L; AUC＞10 (mmol/L × h): the incremental area under curve of glucose>10.0mmol/L.

Supplementary figure 2: Comparison of treatment effects between the Ebenatide and placebo groups from baseline to Week 24

|  | Ebenatide group（N=52） | | Placebo group（N=24） | | *P* |
| --- | --- | --- | --- | --- | --- |
|  | Baseline | Week 24 | Baseline | Week 24 |  |
| SP(mmHg) | 132.17 ± 13.34 | 125.31 ± 10.58 | 130.46 ± 15.93 | 128.87 ± 11.83 | 0.225 |
| DP (mmHg) | 86.00（83.00-90.00） | 82.00（76.00-86.50） | 83.00（80.50-87.00） | 81.50（78.00-84.00） | 0.122 |
| ALT(U/L) | 28.00（20.00-44.25） | 23.10（15.60-32.10） | 27.50（21.28-36.25） | 23.05（18.98-28.63） | 0.757 |
| AST(U/L) | 22.00（14.00-26.18） | 18.20（14.00-21.50） | 18.50（15.25-22.75） | 19.80（15.60-21.48） | 0.568 |
| TC(mmol/l) | 4.86 ± 0.69 | 4.81 ± 0.77 | 4.68 ± 0.71 | 4.70 ± 0.61 | 0.417 |
| TG(mmol/l) | 1.90（1.32-2.74） | 1.76（1.13-2.50） | 1.94（1.44-2.92） | 1.45（1.19-2.63） | 0.386 |
| HDL(mmol/l) | 1.19 ± 0.29 | 1.23 ± 0.31 | 1.15 ± 0.22 | 1.25 ± 0.25 | 0.216 |
| LDL(mmol/l) | 2.91 ± 0.63 | 2.91 ± 0.68 | 2.77 ± 0.56 | 2.88 ± 0.54 | 0.431 |
| Cr (μmol/L) | 66.32 ± 15.73 | 68.03 ± 17.96 | 63.31 ± 10.94 | 65.63 ± 13.37 | 0.507 |
| Hb(g/L) | 151.37 ± 11.94 | 145.92 ± 12.29 | 149.96 ± 17.68 | 149.04 ± 16.71 | 0.044* |
| AMY(U/L) | 57.98 ± 13.87 | 67.79 ± 17.17 | 62.33 ± 15.52 | 70.25 ± 19.74 | 0.433 |
| LIP (U/L) | 82.00（56.50-113.00） | 95.50（70.50-148.75） | 105.00（67.25-133.75） | 111.00（62.00-150.25） | 0.020* |
| FBG(mmol/l) | 8.59 ± 1.64 | 7.94 ± 1.50 | 8.84 ± 1.60 | 8.40 ± 1.07 | 0.274 |
| FCP(ng/ml) | 0.91（0.75-1.14） | 0.86（0.75-1.09） | 0.99（0.74-1.23） | 0.99（0.74-1.32） | 0.610 |
| TyG | 9.46 ± 0.56 | 9.28 ± 0.60 | 9.55 ± 0.56 | 9.33 ± 0.47 | 0.702 |
| HbA1c(%) | 8.48 ± 1.13 | 7.32 ± 0.89 | 8.06 ± 0.76 | 7.47 ± 0.69 | 0.011* |
| Weight (kg) | 72.58 ± 9.94 | 71.52 ± 10.07 | 78.27 ± 15.23 | 77.69 ± 15.48 | 0.464 |
| BMI(kg/m²) | 25.87 ± 2.70 | 25.56 ± 2.58 | 27.90 ± 4.03 | 27.46 ± 4.02 | 0.641 |
| PBF(%) | 29.40 ± 6.11 | 28.00 ± 6.63 | 32.06 ± 6.72 | 31.01 ± 5.84 | 0.624 |
| VFA(cm²) | 98.57 ± 25.27 | 91.89 ± 25.63 | 113.24 ± 38.00 | 106.44 ± 32.18 | 0.912 |
| BFM(kg) | 21.06 ± 5.03 | 19.87 ± 5.33 | 24.71 ± 7.88 | 23.66 ± 6.95 | 0.830 |
| SLM(kg) | 47.72 ± 6.33 | 48.12 ± 6.57 | 48.15 ± 10.17 | 48.28 ± 9.91 | 0.570 |
| FFM(kg) | 50.44 ± 6.67 | 50.89 ± 6.90 | 50.89 ± 10.69 | 51.07 ± 10.41 | 0.590 |
| SMM(kg) | 27.90 ± 4.00 | 28.13 ± 4.17 | 28.15 ± 6.38 | 28.22 ± 6.27 | 0.608 |
| FMI(kg/m²) | 7.72 ± 2.25 | 7.28 ± 2.33 | 9.06 ± 2.70 | 8.61 ± 2.37 | 0.974 |
| SMI(kg/m²) | 7.50（7.10-8.20） | 7.60（7.00-8.20） | 8.10（6.45-9.13） | 7.90（6.45-8.90） | 0.338 |
| WC(cm) | 92.49 ± 7.35 | 90.18 ± 7.18 | 93.63 ± 10.38 | 92.25 ± 10.04 | 0.202 |
| HC(cm) | 97.51 ± 3.71 | 97.12 ± 3.70 | 100.51 ± 7.10 | 100.03 ± 6.91 | 0.800 |
| WHtR | 0.55 ± 0.05 | 0.54 ± 0.05 | 0.56 ± 0.05 | 0.55 ± 0.04 | 0.512 |
| MBG(mmol/l) | 10.52（8.54-12.32） | 8.21（7.49-9.42） | 10.13（9.24-11.26） | 8.86（7.96-9.88） | 0.164 |
| SD(mmol/l) | 2.07（1.70-2.39） | 1.77（1.10-2.40） | 1.89（1.48-2.51） | 1.89（1.47-2.27） | 0.161 |
| CV(%) | 20.40 ± 5.76 | 21.31 ± 8.39 | 19.10 ± 5.10 | 21.71 ± 7.50 | 0.360 |
| TIR(%) | 42.71（16.84-77.26） | 81.94（63.19-97.92） | 58.68（39.41-72.92） | 70.49（60.76-83.33） | 0.031* |
| TBR(%) | 0.00（0.00-0.00） | 0.00（0.00-0.00） | 0.00（0.00-0.00） | 0.00（0.00-0.00） | 0.966 |
| TAR(%) | 57.29（22.74-83.16） | 14.93（2.08-36.63） | 41.32（27.08-60.59) | 29.51（16.67-39.24） | 0.024* |
| AUC＜3.9  (mmol/L × h) | 0.00（0.00-0.00） | 0.00（0.00-0.00） | 0.00（0.00-0.00） | 0.00（0.00-0.00） | 0.977 |
| AUC＞10  (mmol/L × h) | 1554.50（362.50-3904.00） | 378.00（8.50-1188.50） | 1157.00（391.50-2617.50） | 590.00（303.00-1135.00） | 0.060 |

∗*P*<0.05, ∗∗*P*<0.01; SP (mmHg): systolic pressure; DP (mmHg): diastolic pressure; ALT (U/L): alanine aminotransferase; AST (U/L): aspartate aminotransferase; TC (mmol/l): total cholesterol; TG (mmol/l): triglycerides; HDL (mmol/l): high-density lipoprotein; LDL (mmol/l): low-density lipoprotein; Cr (μmol/L): creatinine; Hb (g/L): hemoglobin; AMY (U/L): amylase; LIP (U/L): lipase; FBG (mmol/l): fasting blood glucose; FCP (ng/ml): fasting C-peptide; TyG: triglyceride-glucose index; HbA1c (%): glycated hemoglobin A1c; BMI (kg/m²): body mass index; PBF (%): percent body fat; VFA (cm²): visceral fat area; BFM (kg): body fat mass; SLM (kg): skeletal lean mass; FFM (kg): fat free mass; SMM (kg): skeletal muscle mass; FMI (kg/m²): fat mass index; SMI (kg/m²): skeletal muscle mass index; WC (cm): waist circumference; HC (cm): hip circumference; WHtR: waist-to-height ratio; MBG (mmol/l): mean blood glucose; SD (mmol/l): standard deviation; CV (%): coefficient of variation; TIR (%): percentage of time with glucose levels within 3.9-10.0 mmol/L (target range); TBR (%): percentage of time <3.9 mmol/L; TAR (%): percentage of time >10.0 mmol/L; AUC＜3.9 (mmol/L × h): the incremental area under curve of glucose＜3.9mmol/L; AUC＞10 (mmol/L × h): the incremental area under curve of glucose>10.0mmol/L.

Supplementary figure 3: Comparison of treatment effects between the Ebenatide and placebo groups from baseline to the Week 52

|  | Ebenatide group（N=52） | | Placebo group（N=24） | | *P* |
| --- | --- | --- | --- | --- | --- |
|  | Baseline | Week 52 | Baseline | Week 52 |  |
| SP(mmHg) | 132.17 ± 13.34 | 124.90 ± 14.96 | 130.46 ± 15.93 | 122.00 ± 11.48 | 0.661 |
| DP (mmHg) | 86.00（83.00-90.00） | 82.00（74.00-86.00) | 83.00（80.50-87.00） | 81.00（76.50-84.50) | 0.414 |
| ALT(U/L) | 28.00（20.00-44.25） | 25.85（18.25-37.78) | 27.50（21.28-36.25） | 26.25（18.05-37.00) | 0.724 |
| AST(U/L) | 22.00（14.00-26.18） | 19.40（14.88-26.20) | 18.50（15.25-22.75） | 18.55（15.88-23.28) | 0.956 |
| TC(mmol/l) | 4.86 ± 0.69 | 4.70 ± 0.90 | 4.68 ± 0.71 | 4.20 ± 0.81 | 0.311 |
| TG(mmol/l) | 1.90（1.32-2.74） | 1.90（1.46-2.47） | 1.94（1.44-2.92） | 1.73（1.22-2.23） | 0.199 |
| HDL(mmol/l) | 1.19 ± 0.29 | 1.20 ± 0.32 | 1.15 ± 0.22 | 1.18 ± 0.19 | 0.376 |
| LDL(mmol/l) | 2.91 ± 0.63 | 2.88 ± 0.69 | 2.77 ± 0.56 | 2.64 ± 0.78 | 0.694 |
| Cr (μmol/L) | 66.32 ± 15.73 | 66.28 ± 15.60 | 63.31 ± 10.94 | 66.08 ± 13.64 | 0.131 |
| Hb(g/L) | 151.37 ± 11.94 | 143.72 ± 12.20 | 149.96 ± 17.68 | 146.08 ± 19.12 | 0.147 |
| AMY(U/L) | 57.98 ± 13.87 | 69.00 ± 16.17 | 62.33 ± 15.52 | 79.00 ± 19.09 | 0.175 |
| LIP (U/L) | 82.00（56.50-113.00） | 107.00（74.00-159.00） | 105.00（67.25-133.75） | 156.00（92.00-188.00） | 0.035* |
| FBG(mmol/l) | 8.59 ± 1.64 | 8.36 ± 1.66 | 8.84 ± 1.60 | 8.12 ± 1.52 | 0.223 |
| FCP(ng/ml) | 0.91（0.75-1.14） | 0.94（0.75-1.14） | 0.99（0.74-1.23） | 1.07（0.69-1.31） | 0.606 |
| TyG | 9.46 ± 0.56 | 8.76 ± 0.62 | 9.55 ± 0.56 | 8.69 ± 0.43 | 0.296 |
| HbA1c(%) | 8.48 ± 1.13 | 7.30 ± 0.91 | 8.06 ± 0.76 | 6.85 ± 0.67 | 0.894 |
| Weight (kg) | 72.58 ± 9.94 | 70.72 ± 9.48 | 78.27 ± 15.23 | 76.44 ± 15.64 | 0.968 |
| BMI(kg/m²) | 25.87 ± 2.70 | 25.42 ± 2.57 | 27.90 ± 4.03 | 27.24 ± 4.24 | 0.593 |
| PBF(%) | 29.40 ± 6.11 | 29.09 ± 6.77 | 32.06 ± 6.72 | 31.25 ± 6.17 | 0.602 |
| VFA(cm²) | 98.57 ± 25.27 | 93.57 ± 25.55 | 113.24 ± 38.00 | 106.47 ± 34.52 | 0.678 |
| BFM(kg) | 21.06 ± 5.03 | 20.53 ± 5.62 | 24.71 ± 7.88 | 23.70 ± 7.61 | 0.602 |
| SLM(kg) | 47.72 ± 6.33 | 47.43 ± 6.81 | 48.15 ± 10.17 | 47.62 ± 10.25 | 0.928 |
| FFM(kg) | 50.44 ± 6.67 | 49.85 ± 7.07 | 50.89 ± 10.69 | 50.38 ± 10.75 | 0.889 |
| SMM(kg) | 27.90 ± 4.00 | 27.51 ± 4.22 | 28.15 ± 6.38 | 27.77 ± 6.42 | 0.981 |
| FMI(kg/m²) | 7.72 ± 2.25 | 7.50 ± 2.41 | 9.06 ± 2.70 | 8.61 ± 2.52 | 0.510 |
| SMI(kg/m²) | 7.50（7.10-8.20） | 7.60（6.90-8.20） | 8.10（6.45-9.13） | 7.65（6.18-9.10） | 0.427 |
| WC(cm) | 92.49 ± 7.35 | 90.00 ± 6.79 | 93.63 ± 10.38 | 91.76 ± 10.69 | 0.554 |
| HC(cm) | 97.51 ± 3.71 | 97.02 ± 3.73 | 100.51 ± 7.10 | 99.74 ± 7.47 | 0.643 |
| WHtR | 0.55 ± 0.05 | 0.54 ± 0.05 | 0.56 ± 0.05 | 0.55 ± 0.05 | 0.915 |
| MBG(mmol/l) | 10.52（8.54-12.32） | 8.17（7.35-8.75） | 10.13（9.24-11.26） | 6.91（6.36-8.38） | 0.459 |
| SD(mmol/l) | 2.07（1.70-2.39） | 1.44（1.22-1.91） | 1.89（1.48-2.51） | 1.40（0.94-1.67） | 0.289 |
| CV(%) | 20.40 ± 5.76 | 18.22 ± 4.92 | 19.10 ± 5.10 | 17.67 ± 6.52 | 0.582 |
| TIR(%) | 42.71（16.84-77.26） | 91.67（77.43-97.21) | 58.68（39.41-72.92） | 98.61（81.94-100.00） | 0.310 |
| TBR(%) | 0.00（0.00-0.00） | 0.00（0.00-0.00） | 0.00（0.00-0.00） | 0.00（0.00-0.00） | 0.385 |
| TAR(%) | 57.29（22.74-83.16） | 8.33（2.79-22.57） | 41.32（27.08-60.59) | 1.39（0.00-18.06) | 0.314 |
| AUC＜3.9  (mmol/L × h) | 0.00（0.00-0.00） | 0.00（0.00-0.00） | 0.00（0.00-0.00） | 0.00（0.00-0.00） | 0.385 |
| AUC＞10  (mmol/L × h) | 1554.50（362.50-3904.00） | 116.25（12.38-540.13） | 1157.00（391.50-2617.50） | 5.50（0.00-311.25） | 0.104 |

∗*P*<0.05, ∗∗*P*<0.01; SP (mmHg): systolic pressure; DP (mmHg): diastolic pressure; ALT (U/L): alanine aminotransferase; AST (U/L): aspartate aminotransferase; TC (mmol/l): total cholesterol; TG (mmol/l): triglycerides; HDL (mmol/l): high-density lipoprotein; LDL (mmol/l): low-density lipoprotein; Cr (μmol/L): creatinine; Hb (g/L): hemoglobin; AMY (U/L): amylase; LIP (U/L): lipase; FBG (mmol/l): fasting blood glucose; FCP (ng/ml): fasting C-peptide; TyG: triglyceride-glucose index; HbA1c (%): glycated hemoglobin A1c; BMI (kg/m²): body mass index; PBF (%): percent body fat; VFA (cm²): visceral fat area; BFM (kg): body fat mass; SLM (kg): skeletal lean mass; FFM (kg): fat free mass; SMM (kg): skeletal muscle mass; FMI (kg/m²): fat mass index; SMI (kg/m²): skeletal muscle mass index; WC (cm): waist circumference; HC (cm): hip circumference; WHtR: waist-to-height ratio; MBG (mmol/l): mean blood glucose; SD (mmol/l): standard deviation; CV (%): coefficient of variation; TIR (%): percentage of time with glucose levels within 3.9-10.0 mmol/L (target range); TBR (%): percentage of time <3.9 mmol/L; TAR (%): percentage of time >10.0 mmol/L; AUC＜3.9 (mmol/L × h): the incremental area under curve of glucose＜3.9mmol/L; AUC＞10 (mmol/L × h): the incremental area under curve of glucose>10.0mmol/L.

Supplementary figure 4: Comparison of treatment effects between the Ebenatide and placebo groups from the Week 24 to Week 52

|  | Ebenatide group（N=52） | | Placebo group（N=24） | | *P* |
| --- | --- | --- | --- | --- | --- |
|  | Week 24 | Week 52 | Week 24 | Week 52 |  |
| SP(mmHg) | 125.31 ± 10.58 | 124.90 ± 14.96 | 128.87 ± 11.83 | 122.00 ± 11.48 | 0.046* |
| DP (mmHg) | 82.00（76.00-86.50） | 82.00（74.00-86.00) | 81.50（78.00-84.00） | 81.00（76.50-84.50) | 0.443 |
| ALT(U/L) | 23.10（15.60-32.10） | 25.85（18.25-37.78) | 23.05（18.98-28.63） | 26.25（18.05-37.00) | 0.860 |
| AST(U/L) | 18.20（14.00-21.50） | 19.40（14.88-26.20) | 19.80（15.60-21.48） | 18.55（15.88-23.28) | 0.637 |
| TC(mmol/l) | 4.81 ± 0.77 | 4.70 ± 0.90 | 4.70 ± 0.61 | 4.20 ± 0.81 | 0.125 |
| TG(mmol/l) | 1.76（1.13-2.50） | 1.90（1.46-2.47） | 1.45（1.19-2.63） | 1.73（1.22-2.23） | 0.259 |
| HDL(mmol/l) | 1.23 ± 0.31 | 1.20 ± 0.32 | 1.25 ± 0.25 | 1.18 ± 0.19 | 0.436 |
| LDL(mmol/l) | 2.91 ± 0.68 | 2.88 ± 0.69 | 2.88 ± 0.54 | 2.64 ± 0.78 | 0.320 |
| Cr (μmol/L) | 68.03 ± 17.96 | 66.28 ± 15.60 | 65.63 ± 13.37 | 66.08 ± 13.64 | 0.135 |
| Hb(g/L) | 145.92 ± 12.29 | 143.72 ± 12.20 | 149.04 ± 16.71 | 146.08 ± 19.12 | 0.752 |
| AMY(U/L) | 67.79 ± 17.17 | 69.00 ± 16.17 | 70.25 ± 19.74 | 79.00 ± 19.09 | 0.032* |
| LIP (U/L) | 95.50（70.50-148.75） | 107.00（74.00-159.00） | 111.00（62.00-150.25） | 156.00（92.00-188.00） | 0.005** |
| FBG(mmol/l) | 7.94 ± 1.50 | 8.36 ± 1.66 | 8.40 ± 1.07 | 8.12 ± 1.52 | 0.036* |
| FCP(ng/ml) | 0.86（0.75-1.09） | 0.94（0.75-1.14） | 0.99（0.74-1.32） | 1.07（0.69-1.31） | 0.812 |
| TyG | 9.28 ± 0.60 | 8.76 ± 0.62 | 9.33 ± 0.47 | 8.69 ± 0.43 | 0.465 |
| HbA1c(%) | 7.32 ± 0.89 | 7.30 ± 0.91 | 7.47 ± 0.69 | 6.85 ± 0.67 | ＜0.001** |
| Weight (kg) | 71.52 ± 10.07 | 70.72 ± 9.48 | 77.69 ± 15.48 | 76.44 ± 15.64 | 0.513 |
| BMI(kg/m²) | 25.56 ± 2.58 | 25.42 ± 2.57 | 27.46 ± 4.02 | 27.24 ± 4.24 | 0.794 |
| PBF(%) | 28.00 ± 6.63 | 29.09 ± 6.77 | 31.01 ± 5.84 | 31.25 ± 6.17 | 0.359 |
| VFA(cm²) | 91.89 ± 25.63 | 93.57 ± 25.55 | 106.44 ± 32.18 | 106.47 ± 34.52 | 0.814 |
| BFM(kg) | 19.87 ± 5.33 | 20.53 ± 5.62 | 23.66 ± 6.95 | 23.70 ± 7.61 | 0.474 |
| SLM(kg) | 48.12 ± 6.57 | 47.43 ± 6.81 | 48.28 ± 9.91 | 47.62 ± 10.25 | 0.662 |
| FFM(kg) | 50.89 ± 6.90 | 49.85 ± 7.07 | 51.07 ± 10.41 | 50.38 ± 10.75 | 0.485 |
| SMM(kg) | 28.13 ± 4.17 | 27.51 ± 4.22 | 28.22 ± 6.27 | 27.77 ± 6.42 | 0.568 |
| FMI(kg/m²) | 7.28 ± 2.33 | 7.50 ± 2.41 | 8.61 ± 2.37 | 8.61 ± 2.52 | 0.490 |
| SMI(kg/m²) | 7.60（7.00-8.20） | 7.60（6.90-8.20） | 7.90（6.45-8.90） | 7.65（6.18-9.10） | 0.902 |
| WC(cm) | 90.18 ± 7.18 | 90.00 ± 6.79 | 92.25 ± 10.04 | 91.76 ± 10.69 | 0.532 |
| HC(cm) | 97.12 ± 3.70 | 97.02 ± 3.73 | 100.03 ± 6.91 | 99.74 ± 7.47 | 0.703 |
| WHtR | 0.54 ± 0.05 | 0.54 ± 0.05 | 0.55 ± 0.04 | 0.55 ± 0.05 | 0.659 |
| MBG(mmol/l) | 8.21（7.49-9.42） | 8.17（7.35-8.75） | 8.86（7.96-9.88） | 6.91（6.36-8.38） | 0.149 |
| SD(mmol/l) | 1.77（1.10-2.40） | 1.44（1.22-1.91） | 1.89（1.47-2.27） | 1.40（0.94-1.67） | 0.845 |
| CV(%) | 21.31 ± 8.39 | 18.22 ± 4.92 | 21.71 ± 7.50 | 17.67 ± 6.52 | 0.946 |
| TIR(%) | 81.94（63.19-97.92） | 91.67（77.43-97.21) | 70.49（60.76-83.33） | 98.61（81.94-100.00） | 0.447 |
| TBR(%) | 0.00（0.00-0.00） | 0.00（0.00-0.00） | 0.00（0.00-0.00） | 0.00（0.00-0.00） | 0.146 |
| TAR(%) | 14.93（2.08-36.63） | 8.33（2.79-22.57） | 29.51（16.67-39.24） | 1.39（0.00-18.06) | 0.330 |
| AUC＜3.9  (mmol/L × h) | 0.00（0.00-0.00） | 0.00（0.00-0.00） | 0.00（0.00-0.00） | 0.00（0.00-0.00） | 0.146 |
| AUC＞10  (mmol/L × h) | 378.00（8.50-1188.50） | 116.25（12.38-540.13） | 590.00（303.00-1135.00） | 5.50（0.00-311.25） | 0.761 |

∗*P*<0.05, ∗∗*P*<0.01; SP (mmHg): systolic pressure; DP (mmHg): diastolic pressure; ALT (U/L): alanine aminotransferase; AST (U/L): aspartate aminotransferase; TC (mmol/l): total cholesterol; TG (mmol/l): triglycerides; HDL (mmol/l): high-density lipoprotein; LDL (mmol/l): low-density lipoprotein; Cr (μmol/L): creatinine; Hb (g/L): hemoglobin; AMY (U/L): amylase; LIP (U/L): lipase; FBG (mmol/l): fasting blood glucose; FCP (ng/ml): fasting C-peptide; TyG: triglyceride-glucose index; HbA1c (%): glycated hemoglobin A1c; BMI (kg/m²): body mass index; PBF (%): percent body fat; VFA (cm²): visceral fat area; BFM (kg): body fat mass; SLM (kg): skeletal lean mass; FFM (kg): fat free mass; SMM (kg): skeletal muscle mass; FMI (kg/m²): fat mass index; SMI (kg/m²): skeletal muscle mass index; WC (cm): waist circumference; HC (cm): hip circumference; WHtR: waist-to-height ratio; MBG (mmol/l): mean blood glucose; SD (mmol/l): standard deviation; CV (%): coefficient of variation; TIR (%): percentage of time with glucose levels within 3.9-10.0 mmol/L (target range); TBR (%): percentage of time <3.9 mmol/L; TAR (%): percentage of time >10.0 mmol/L; AUC＜3.9 (mmol/L × h): the incremental area under curve of glucose＜3.9mmol/L; AUC＞10 (mmol/L × h): the incremental area under curve of glucose>10.0mmol/L.
